# Paraphenylenediamine (PPD) Poisoning Mistaken for an Anaphylactic Reaction

**DOI:** 10.7759/cureus.22503

**Published:** 2022-02-22

**Authors:** Mohamed Elgassim, Khalid Y Fadul, Mohammed Abbas, Faten AlBakri, Raghav Kamath, Waleed Salem

**Affiliations:** 1 Emergency Medicine, Hamad General Hospital, Doha, QAT; 2 Internal Medicine, Hamad General Hospital, Doha, QAT; 3 Emergency Medicine, Hamad Medical Corporation, Doha, QAT

**Keywords:** female gender, pregnant and lactating, suicide attempts, poison, self poisoning, black henna tattoo, henna

## Abstract

Paraphenylenediamine (PPD) is commonly used in parts of Africa and Asia in combination with Lawsonia alba leaves (also widely known as henna) or as a substitute to dye the hair, palms, soles, or arms for wedding ceremonies or spiritual events. At the same time, it is quickly trending as an agent for suicidal attempts through ingestion. Toxicity is dose-dependent and can lead to serious complications both locally, such as angioedema and airway swelling, or systemically such as acute kidney injury, fatal arrhythmias, and acute hepatitis.

We present a case of a 26-year-old pregnant female patient, with no known underlying medical history or known allergies. She ingested PPD-based dye in an attempt to end her life. She initially presented asymptomatically but started developing delayed toxicity symptoms including angioedema and acute liver failure. Her initial diagnosis was an anaphylaxis reaction, and her workup and management were conducted accordingly.

We discuss the appropriate course of action in terms of investigations and management in cases of PPD poisoning, and what measures should have been taken in this patient to provide the best healthcare outcome.

## Introduction

Paraphenylenediamine (PPD) is widely used as an oxidizable hair dye that is well established to be a cause of skin irritation and dermatitis in susceptible individuals [1]. When ingested, it causes severe local irritation and edema of the face and neck and systemic manifestations of ingestions include rhabdomyolysis and acute renal failure, which may be fatal if not treated aggressively [2,3]. Intentional ingestion of PPD as a suicidal attempt is frequently reported from Africa and the Middle East [4,5]. In most cases presented in the literature, the progression of clinical symptoms appears the same with some variations. So, even though there is no specific laboratory test to aid with the diagnosis of this poisoning, knowledge of the common features of PPD poisoning can help limit the morbidities and possible mortalities.

## Case presentation

We report a case of a 26-year-old female with no known underlying medical illness, surgical history, mental illness, or known allergies. She presented to the emergency department after an intentional ingestion of Paraphenylenediamine (PPD) based dye. The dye was in the form of granules and the amount she ingested was unknown, however, estimation by the toxicology team was approximately 5mg. The ingestion occurred at around 1200hrs, and her husband immediately called the ambulance. At the time, she was vitally stable with no sign of distress. She remained so even after her arrival at the emergency department for observation and monitoring. Her initial labs were showing normal levels of complete blood count (CBC) parameters, however, marked elevation in creatine kinase (CK) at >22,000 and myoglobin of 5,969 both indicative of rhabdomyolysis. Her liver enzymes levels at the time were hemolyzed. At 1700hrs she developed multiple episodes of vomiting, shortness of breath, and tightness in the mouth. Upon reassessment, she was tachypneic with 36 breaths per minute, tachycardic at 107 beats minute, normal blood pressure at 108/78 mmHg, and was maintaining 99% saturation on room air. Her examination revealed swelling in her tongue and the floor of her mouth, however, her chest was clear with good air entry bilaterally and no stridor. Repeat labs showed CK of 1052 (Normal value: 26-129), myoglobin of 1650 (Normal value: 25-58), alanine transaminase (ALT) of 221 (Normal value: 0-33), and aspartate transaminase (AST) of 1,220 (Normal value: 0-32). The patient was shifted to the high acuity area where she received epinephrine 1mg IM, epinephrine 4mg nebulization, dexamethasone 8mg IV, and metoclopramide 10mg IV. At 0200hrs, there was an improvement in hemodynamic parameters and she was planned for 24 hours of observation and was therefore shifted to the observation unit. The toxicology team had assessed her, and she was feeling better than the day before. Consultation of psychiatry services revealed that the ingestion was an attempt to end her own life after getting pregnant out of wedlock during her recent travels and was six weeks pregnant. Obstetrician on call consulted after 24 hours from the presentation and recommended sending for beta-HCG levels and to refer the patient to the Women's Wellness and Research Center after stabilization. However, she was still having difficulty swallowing, significant mandibular swelling, and had trismus of about 1.5cm. She was started on hydrocortisone 100mg IV, electrocardiography and venous blood gases were requested Q6hrly and blood works Q12hrly for optimum monitoring. Ear, nose, and throat (ENT) team was consulted and agreed to admit the patient for 24 hours of observation under their unit. The fiberoptic assessment determined that there was no airway compromise. After her observation period ended, she was transferred into the mental health hospital, where her husband discharged her against medical advice within a few hours of admission.

## Discussion

Poisoning is a common method of suicide [6]. Paraphenylenediamine (PPD) has been utilized increasingly as a method of poisoning in developing countries over the last decades [7], especially among females [8-9]. There is very scarce reporting in the literature of PPD-related suicide attempts in pregnant females [9].

PPD is a derivative of p-nitroaniline [10] and is commonly used in the Middle East, parts of Africa, India, and Pakistan in combination with Lawsonia alba leaves (also widely known as henna) or as a substitute to dye the hair, palms, soles or arms [10,11]. PPD metabolization produces Bandrowski's base, a highly toxic metabolite [11].

PPD poisoning is dose-dependent [6,10,11] and has high mortality [11]. The lethal dose has not been established yet, but it is estimated to be 7-10g. Large doses can be fatal within the first 24 hours [10]. Clinical presentation of PPD poisoning varies depending on ingestion to presentation time, and ingested amount. It usually manifests as respiratory distress secondary to cervicofacial edema in early presentations. Late presentations manifest as acute kidney injury (AKI) secondary to acute tubular necrosis, and rhabdomyolysis and myoglobin deposition in the kidney. Other less common presentations in the literature include fatal arrhythmias, acute hepatitis, vomiting, gastritis, hypertension, vertigo, tremor, and convulsions [7,9-12] as well as anemia, leukocytosis, hemoglobinuria, and hemoglobinuria. There is at least one case report of liver necrosis [13].

Management is mainly supportive as there is no antidote available yet [6,10]. Angioedema is managed by early intubation or tracheostomy [6,10,11]. As hypersensitivity reactions are still among the initial differential diagnosis, antihistamines and steroids are commonly used as part of initial management. The role of hemodialysis in removing PPD is unclear [6]; however, alkaline diuresis using isotonic saline, sodium bicarbonate, and diuretics are used to manage myoglobinuria [6] which can precipitate and aggravate AKI. Overall, early recognition, timely referral, and adequate supportive therapy improve clinical outcomes [6].

We report a case of a 26-year-old pregnant female presented to the emergency department at six weeks gestational age after ingestion of an unknown amount of PPD-based dye. Her symptoms started 5 hours after ingestion. Significant laboratory results were high CK and liver function tests (LFTs) while renal function tests (RFTs) were normal. She was treated with intravenous fluids, epinephrine, dexamethasone, and metoclopramide. Neither invasive respiratory intervention nor hemodialysis was needed. She was transferred to psychiatry's care after airway compromise was ruled out. The obstetrician on call was consulted after 24 hours. Obstetric ultrasonography done nine weeks later confirmed the viability of the pregnancy. A descriptive study done by Tanweer et al. suggested that the rate of pregnancy loss in PPD poisoning is no more than 16.6% [9].

One of the limitations that we faced with this case is the absence of any documentation of the ECG readings. There is no clear assessment of the heart electrophysiology, although arrhythmias are one of the major complications of PPD ingestion. Another issue was that after the patient was transferred to psychiatry, there was no follow-up on her liver enzymes and no clear assessment of her liver function although she was in acute injury. Lastly, we faced another issue with the abrupt continuation of care, as the patient's husband discharged her against medical advice, and no further follow-up on her physiological status after the toxicity episode was made.

## Conclusions

PPD toxicity has high morbidity and mortality, but early assessment and prompt management can alter the prognosis of these patients. Special considerations should be given to pregnant females, which calls for the early involvement of obstetricians for their input. Abnormal laboratory results need to be followed up as they may indicate organ damage. Early interventions, if that is the case, can prevent permanent complications. The absence of a standardized PPD poisoning protocol can lead to a discrepancy in managing the case, which can cause grave consequences. Practitioners should be aware of the differential diagnosis of anaphylaxis in the face of such ingestion or in a patient with potential exposure. Further clinical or biochemical methods to differentiate or identify this patient population compared to the average patient with anaphylaxis needs to be researched.
